# Association of human papillomavirus type 16 long control region mutation and cervical cancer

**DOI:** 10.1186/1743-422X-10-30

**Published:** 2013-01-23

**Authors:** Chamsai Pientong, Parichat Wongwarissara, Tipaya Ekalaksananan, Piyawut Swangphon, Pilaiwan Kleebkaow, Bunkerd Kongyingyoes, Sumalee Siriaunkgul, Kobkul Tungsinmunkong, Cheepsumon Suthipintawong

**Affiliations:** 1 Department of Microbiology, Faculty of Medicine, Khon Kaen University, Khon Kaen, Thailand; 2 Department of Obstetrics and Gynecology, Faculty of Medicine, Khon Kaen University, Khon Kaen, Thailand; 3 Department of Pharmacology, Faculty of Medicine, Khon Kaen University, Khon Kaen, Thailand; 4 Department of Pathology, Faculty of Medicine, Chiang Mai University, Chiang Mai, Thailand; 5 Institute of Pathology, Department of Medical Services, Ministry of Public Health, Bangkok, Thailand

**Keywords:** HPV16, Sub-lineage Asian, Cervical cancer, Long control region

## Abstract

**Background:**

The variation of human papillomavirus (HPV) genes or HPV variants demonstrates different risks of cervical cancer. Mutation in the long control region (LCR) at YY1-motifs is one of the mechanisms for enhancing viral oncogene expression during the course of cancer cell progression. In Thai women, cervical cancers are almost always associated with HPV16 variant sub-lineage Asian (HPV16As); however, the mechanism involved remains elusive. The aim of this study was to understand further the oncogenic potential of HPV16As.

**Methods:**

A total of 82 HPV16-positive specimens from Thai women were selected from formalin-fixed paraffin-embedded cervical tissues, and the full length E6 gene of each specimen was amplified and sequenced. LCRs of the HPV16As-positive cases were amplified and sequenced to analyze their polymorphisms. Transcriptional activities of the HPV16As LCRs were then compared with sub-lineage European (EUR), sub-lineage Asian-American 1 (AA1) and HPV16 prototype by insertion of the LCRs into the pGL3-Basic vector.

**Results:**

The HPV16 DNA sequences were classified as HPV16 prototype (18.3%), Asian (As, 61%), Asian American-1 (AA1, 8.5%), European (EUR, 7.3%), Asian African-2 (AFR2, 3.7%) and Java-135C (J135C, 1.2%). The prevalence of HPV16As was 30% in low-grade squamous intraepithelial lesion (LSIL), while that in high-grade squamous intraepithelial lesion (HSIL) and squamous cell cervical carcinoma (SCC) were 63.9% and 66.7%, respectively, which demonstrates a significant association of HPV16As with the disease severity. LCR polymorphisms from 43 HPV16As positive cases were analyzed by PCR-sequencing. Thirty-eight nucleotide variation positions spanned nucleotide positions 7157–82. Ten new mutations found in the HPV16As LCRs were located predominantly at the enhancer and proximal to the 3’-end of the early promoter. The LCRs of the common HPV16As, EUR and AA1 showed 5, 13 and 23-fold higher activity than the HPV16 prototype LCR, while those of the new nucleotide variations of As showed 19 (As-sv1) and 30 (As-sv14) -fold higher activity than the HPV16 prototype.

**Conclusions:**

HPV16As DNA sequence variation, especially at the proximal to early promoter in the LCR, enhances transcriptional activity. This could be one of the possible mechanisms for HPV16As-associated cervical cancer development.

## Background

Cervical cancer is the second most common cancer among women worldwide, and it remains a leading cause of cancer-related death in women in developing countries [1]. The most important risk factor that is associated with cervical cancer is infection with high-risk human papillomaviruses (HR-HPVs), particularly HPV16, which is the most prevalent type and is found in approximately 55% of individuals with cervical cancer [1,2]. Previous studies have shown that nucleotide alterations in HPV16 affect the oncogenic potential of the virus [3]. The first worldwide study of HPV16 variants was performed by Ho et al. in 1993 [4]. The study reported that HPV16 long control region (LCR) variants robustly segregate into a phylogenetic tree with five major variant lineages that are named based on the geographical origin of the population. Yamada et al. [2] used sequence analysis of several genes, including the E6 open reading frame (ORF), to demonstrate that HPV16 variants can be classified into six major variant lineages: European (EUR), Asian (As), Asian American (AA), African-1 (AFR-1), African-2 (AFR-2) and North American (NA). This analysis suggested that 26% of Southeast Asian tissue samples contain the As variant lineage, which is rare or absent in other continents. Cornet et al. [5] confirmed variant lineages and sub-classifications that provide nine HPV16 variant sub-lineages (EUR, As, AFR1a, AFR1b, AFR2a, AFR2b, NA, AA1 and AA2) based upon 13 and 32 phylogenetically distinct positions in E6 and LCR, respectively.

Infection with HR-HPV induces carcinogenesis through dysregulation of E6 and E7 oncogene expression, which is controlled by the p97 early promoter that is located in the E6 proximal part of the LCR. The HPV16 LCR is approximately 850 bp and contains the early promoter and various transcriptional regulatory motifs for both viral and host proteins [6].

Transcriptional analysis of the HPV16 LCR is important to evaluate the association between LCR sequence variation and the oncogenic potential of HPV16 variants. Sequence variations in the LCR of HPV16 may modulate the oncogenic potential of HPV16, as nucleotide changes within this regulatory region influence replication and transcription through their effect on regulatory protein complex formation on DNA [7]. A special role has been assigned to variations that occur within YY1 binding sites in the LCR, as these variations were found to enhance expression of the viral oncoproteins [8]. Several groups have reported different activities of HPV16 variants and, as a result, have classified the variants into geographically clustered phylogenetic groups [9-11].

Infection with HPV16 variant sub-lineage Asian (HPV16As) is found predominantly in cervical cancer cases with HPV16 positive (73.9%) in Khon Kean, Thailand [12], yet data regarding the transcriptional activity of HPV16As are absent. In the present study, we investigated the association between the risk of cervical cancer and the genetic variations of HPV16. For this purpose, we analyzed the genetic polymorphisms in the LCR of HPV16As. In addition, transcriptional activity of LCRs from 4 DNA samples of HPV16As was examined and compared to AA1, EUR and HPV16 prototype to establish the role of infection in cervical carcinogenesis.

## Results

### The association of cervical cancer risk and HPV16As

All 82 HPV16-positive samples from Thai women were investigated for HPV16 variant lineages or sub-lineages according to Yamada’s classification using specific primer sets for the HPV16 E6 gene. The most common HPV16 variant sub-lineage was As (61%). The proportion of HPV16As increased proportionally (OR 4.387; 95% CI 1.043-18.457; *P* = 0.042) with the severity of cervical lesions (30% of LSIL, 63.9% of HSIL and 66.7% of SCC) (Table 1).

**Table 1:** **HPV16 variant sub**-**lineages in each cervical lesion**

**Sub**-**lineages**	**LSIL** (%)	**HSIL** (%)	**SCC** (%)	**Total** (%)
Prototype	5 (50)	6 (16.7)	4 (11.1)	15 (18.3)
As	3 (30)	23 (63.9)	24 (66.7)	50 (61)
AA1	-	2 (5.5)	5 (13.9)	7 (8.5)
EUR	2 (20)	4 (11.1)	-	6 (7.3)
AFR2	-	1 (2.8)	2 (5.5)	3 (3.7)
J135C	-	-	1 (2.8)	1 (1.2)
Total	10	36	36	82 (100)

LSIL = low-grade squamous intraepithelial lesion.

HSIL = high-grade squamous intraepithelial lesion.

SCC = squamous cell cervical carcinoma.

### Polymorphisms of LCRs among HPV16As

To investigate the mutations in the LCRs among HPV16As, the 47 DNA samples from HPV16As- positive cases with HSIL and SCC were studied. The HPV16As LCRs from 43 cases were successfully amplified and sequenced. Blast analysis was performed against HPV16 prototype LCR sequences [GenBank:AY686584] to evaluate gene mutations.

A total of 38 nucleotide variation positions was found in a region spanning nucleotide positions 7157–82 in the HPV16As LCR (Table 2). At least 22 of these nucleotide alterations were previously reported by other authors [10,13]. In addition, we found 10 nucleotide variation positions that have not been previously reported, including 7218 T>A, 7384 T>G, 7429 G>A, 7430C>T, 7617C insertion with A, 7844A>C, 7874C>G, 28 G insertion with A (SP-1), 46 T insertion with G (SP-1, E2BS-2) and 61 T insertion with G (E2BS-1). This result shows that the HPV16As polymorphisms have a specific combination of 12 specific nucleotide variations in the LCR, of which six are diagnostic: 7177 T>C, 7201 T>C, 7270C>T, 7287A>C, 7842 G>A/T and 24C>T. According these mutations, 37.2% (18.6% of each SCC and HSIL) were recognized as common HPV16As. HPV16As sub-variant (As-sv) was identified as As-sv1-22 depending on different nucleotide mutations (Table 2).

**Table 2:** **Nucleotide variations in HPV16As LCRs from HSIL and SCC samples compared with the HPV16 LCR reference sequence** [**GenBank**:**AY686584**]

	**HPV16 LCR**																																						**Number of samples** (%) (**n**=**43**)
	1	2	3	4	5	6	7	8	9	10	11	12	13	14	15	16	17	18	19	20	21	22	23	24	25	26	27	28	29	30	31	32	33	34	35	36	37	38	
**Binding site**					**TEF**-**1**													**GRE**-**1**	**GRE**-**1**					**YY**-**1**			**YY**-**1**		**YY**-**1**, **SP**-**1**, **OCT**-**1**		**E2BS**-**3**				**SP**-**1**	**SP**-**1**, **E2BS**-**2**	**E2BS**-**1**		
% **of samples**	100	100	7	2.3	100	100	2.3	2.3	4.7	95.3	100	95.3	4.7	2.3	2.3	41.9	4.7	2.3	2.3	100	2.3	2.3	100	9.3	2.3	2.3	2.3	2.3	100	2.3	2.3	39.5	2.3	100	4.7	4.7	4.7	100	
**Changed position**	7175	7177	7179	7186	7193	7201	7213	7217	^*^7218	7270	7287	7289	^*^7384	7405	7418	^*^7429	^*^7430	7485	7489	7521	^*^7617	7623	7730	7781	7791	7792	7802	7813	7842	^*^7844	7868	^*^7874	7886	24	^*^28	^*^46	^*^61	81	
**HPV16 pt.**	A	T	G	T	G	T	G	G	T	C	A	A	T	T	T	G	C	A	G	G	C	G	A	T	T	C	C	T	G	A	G	C	C	C	G	T	T	G	
**HPV16 As**	C	C	-	-	T	C	-	-	-	-	C	-	-	-	-	-	-	-	-	A	-	-	C	-	-	-	-	-	A/T	-	-	-	-	T	-	-	-	-	
**SCC**																																							
As	C	C	-	-	T	C	-	-	-	T	C	C	-	-	-	-	-	-	-	A	-	-	C	-	-	-	-	-	A	-	-	-	-	T	-	-	-	T	8 (18.6%)
As-sv1	C	C	-	-	T	C	-	-	-	T	C	C	-	-	-	A	-	-	-	A	-	-	C	-	-	-	-	-	A	-	-	G	-	T	-	-	-	T	4 (9.3%)
As-sv2	C	C	-	G	T	C	-	-	-	T	C	C	-	-	-	-	-	-	-	A	-	-	C	C	-	T	-	-	T	-	-	-	-	T	-	-	-	T	1 (2.3%)
As-sv3	C	C	-	-	T	C	-	A	-	T	C	C	-	-	-	-	-	-	-	A	-	C^	C	-	-	-	-	-	A	-	-	-	-	T	-	-	-	T	1 (2.3%)
As-sv4	C	C	-	-	T	C	-	-	A	T	C	-	-	-	-	-	-	-	-	A	-	-	C	-	-	-	-	-	A	-	-	-	-	T	-	-	-	T	1 (2.3%)
As-sv5	C	C	-	-	T	C	-	-	-	T	C	T	G	-	-	-	T	-	-	A	-	-	C	-	-	-	-	-	A	-	-	-	-	T	-	-	-	T	1 (2.3%)
As-sv6	C	C	-	-	T	C	-	-	-	T	C	T	G	-	-	-	T	-	-	A	-	-	C	-	-	-	A	-	A	-	-	-	-	T	-	-	-	T	1 (2.3%)
As-sv7	C	C	-	-	T	C	-	-	-	T	C	-	-	G	-	-	-	-	-	A	-	-	C	-	-	-	-	-	A	-	-	-	-	T	-	-	-	T	1 (2.3%)
As-sv8	C	C	-	-	T	C	-	-	-	T	C	C	-	-	A	A	-	-	-	A	A^	-	C	-	-	-	-	-	A	-	-	G	-	T	-	-	-	T	1 (2.3%)
As-sv9	C	C	-	-	T	C	-	-	-	T	C	C	-	-	-	-	-	-	-	A	-	-	C	C	-	-	-	-	A	-	-	-	-	T	-	-	-	T	1 (2.3%)
As-sv10	C	C	-	-	T	C	-	-	-	T	C	C	-	-	-	-	-	-	-	A	-	-	C	C	-	-	-	-	T	C	-	-	-	T	-	-	-	T	1 (2.3%)
As-sv11	C	C	-	-	T	C	-	-	-	T	C	C	-	-	-	-	-	-	-	A	-	-	C	C	-	-	-	-	A	-	-	G	-	T	-	-	-	T	1 (2.3%)
As-sv12	C	C	-	-	T	C	-	-	-	-	C	C	-	-	-	-	-	C	A	A	-	-	C	-	-	-	-	-	A	-	-	-	-	T	-	-	-	T	1 (2.3%)
As-sv13	C	C	-	-	T	C	-	-	-	T	C	C	-	-	-	A	-	-	-	A	-	-	C	-	-	-	-	-	A	-	-	G	-	T	-	G^	-	T	1 (2.3%)
As-sv14	C	C	-	-	T	C	-	-	-	T	C	C	-	-	-	-	-	-	-	A	-	-	C	-	-	-	-	-	A	-	-	G	-	T	A^	G^	G^	T	1 (2.3%)
As-sv15	C	C	-	-	T	C	-	-	-	T	C	C	-	-	-	-	-	-	-	A	-	-	C	-	-	-	-	-	A	-	-	-	-	T	A^	-	G^	T	1 (2.3%)
As-sv16	C	C	-	-	T	C	-	-	-	T	C	C	-	-	-	A	-	-	-	A	-	-	C	-	del	-	-	G^	A	-	-	-	-	T	-	-	-	T	1 (2.3%)
**HSIL**																																							
As-sv1	C	C	-	-	T	C	-	-	-	T	C	C	-	-	-	A	-	-	-	A	-	-	C	-	-	-	-	-	A	-	-	G	-	T	-	-	-	T	8 (18.6%)
As-sv17	C	C	T	-	T	C	-	-	-	T	C	C	-	-	-	A	-	-	-	A	-	-	C	-	-	-	-	-	A	-	-	-	-	T	-	-	-	T	2 (4.7%)
As-sv18	C	C	-	-	T	C	-	-	-	T	C	C	-	-	-	A	-	-	-	A	-	-	C	-	-	-	-	-	A	-	-	-	-	T	-	-	-	T	2 (4.7%)
As-sv19	C	C	T	-	T	C	C^	-	-	T	C	C	-	-	-	-	-	-	-	A	-	-	C	-	-	-	-	-	A	-	-	G	-	T	-	-	-	T	1 (2.3%)
As-sv20	C	C	-	-	T	C	-	-	A	-	C	C	-	-	-	A	-	-	-	A	-	-	C	-	-	-	-	-	A	-	-	-	-	T	-	-	-	T	1 (2.3%)
As-sv21	C	C	-	-	T	C	-	-	-	T	C	C	-	-	-	A	-	-	-	A	-	-	C	-	-	-	-	-	A	-	A	G	-	T	-	-	-	T	1 (2.3%)
As-sv22	C	C	-	-	T	C	-	-	-	T	C	C	-	-	-	A	-	-	-	A	-	-	C	-	-	-	-	-	A	-	-	G	A	T	-	-	-	T	1 (2.3%)

*Novel nucleotide sequence variation found in this study, HPV16pt. = HPV16 prototype [GenBank:AY686584], As = HPV16As, As-sv = HPV16As sub-variant, TEF-1 = transcription factor binding site, GRE-1 = glucocorticoid response element, YY-1 = yin yang factor, SP-1 = *trans*-acting transcription factor, Oct-1 = octamer binding transcription factor, E2BS = E2 binding site.

Nucleotide sequence alterations were also detected within the binding site of TEF-1 (7193 G>T), YY-1, SP-1 and OCT-1 (7842 G>A or T) in the LCRs of all 43 HPV16As cases (100%). Additionally, five nucleotide changes in HPV16As LCRs were found within known binding sites of GRE-1 (7485A>C and 7489 G>A), YY-1 (7781 T>C and 7802C>A) and E2BS-3 (7868 G>A), with a detection rate of 2.3%, 2.3%, 9.30%, 2.3% and 2.3%, respectively. The novel nucleotide alterations found in this study at positions 7429 G>A and 7874C>G were the most common variations, with a prevalence of 41.9% and 39.5%, respectively. Moreover, 4.7% of the 43 samples exhibited a nucleotide change proximal to the p97 promoter, especially at positions 46 and 61, which correspond to the binding sites for SP-1, E2BS-2 and E2BS-1.

### HPV16As LCR transcriptional activity

To determine the consequences of HPV16As LCR nucleotide sequence variation, the transcriptional activity of HPV16As LCRs was compared with that of HPV16 prototype and the sub-lineages, AA1 and EUR. Nucleotide variations in the LCRs from HPV16As, As-sv1, As-sv14, AA1 and EUR are proximal to the p97 promoter for the E6/E7 oncogenes and are shown in Table 3. The common HPV16As (no. 15 and 36) contains LCR isolated from HSIL and SCC. The HPV16As-sv1 and HPV16As-sv14 contain HPV16As LCR mutations at positions which are reported for the first time in this study as novel variation: 7429 G>A, 7874C>G, 28 G insertion with A (SP-1), 46 T insertion with G (SP-1, E2BS2) and 61 T insertion with G (E2BS1).

**Table 3:** **The positions of nucleotide variation in LCRs from the HPV16 variant sub**-**lineages**

**nt**. **position**	**7177**	**7193**	**7201**	**7270**	**7287**	**7289**	**7429**	**7521**	**7730**	**7842**	**7874**	**24**	**28**	**46**	**61**	**81**
Prototype^a^	T	G	T	C	A	A	G	G	A	G	C	C	G	T	T	G
As^b^ (no. 15)	C	T	C	T	C	C	G	A	C	A	C	T	G	T	T	T
As^b^ (no. 36)	C	T	C	T	C	C	G	A	C	A	C	T	G	T	T	T
As-sv1^c^ (no. 30)	C	T	C	T	C	C	**A**	A	C	A	**G**	T	G	T	T	T
As-sv14^c^ (no. 42)	C	T	C	T	C	C	**A**	A	C	A	**G**	T	**A**^	**G**^	**G**^	T
**nt**. **position**	**7193**	**7233**	**7339**	**7394**	**7395**	**7485**	**7489**	**7521**	**7669**	**7689**	**7729**	**7743**	**7764**	**7786**	**7886**	**28**
Prototype^a^	G	A	A	C	C	A	G	G	C	C	A	T	C	C	C	G
EUR (no. 43)	G	A	A	C	C	A	G	**A**	C	C	A	T	C	C	C	**A**^
AA1 (no. 47)	T	T	T	T	T	C	A	A	T	A	C	G	T	T	G	G

^a^HPV16 prototype LCR, the reference sequence [GenBank:AY686584].

^b^HPV16As LCR, the sequence found in previous studies in other continents.

^c^HPV16As sub-variant LCR, the novel sequence variation reported in this study.

**Figure 1:**
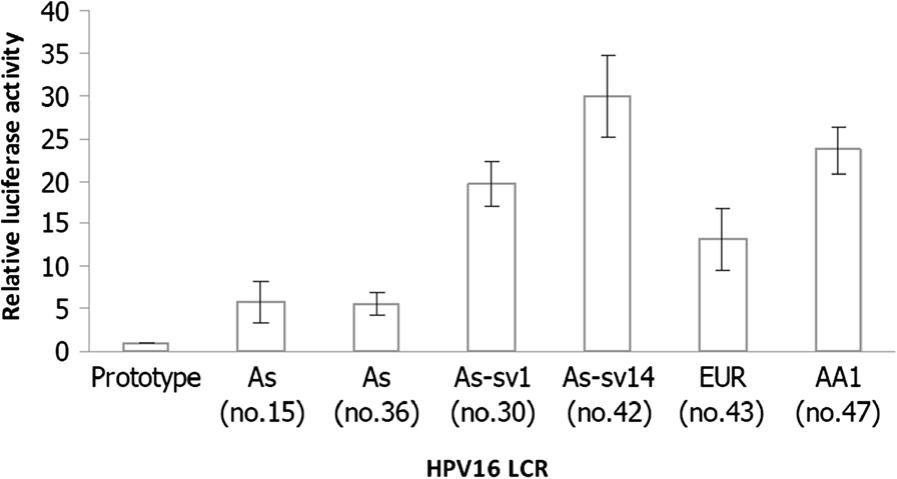
**Transcriptional activity of the LCRs from the HPV16 prototype**, **As**, **As**-**sv1**, **As**-**sv14**, **AA1 and EUR.** The transcriptional activity was determined by luciferase activity in the C33A cell line, and the relative luciferase activity of HPV16As was compared to the prototype. The data presented represent average of at least 3 independent experiments, with error bars indicating standard variations.

Figure 1 shows LCR transcriptional activities, and the results indicate that all of the HPV16As, EUR and AA1 LCRs show higher transcriptional activity than the prototype. Both HPV16As-sv1 and HPV16As-sv14 exhibited higher transcriptional activity than the common HPV16As and the HPV16As-sv14 showed the highest transcriptional activity with 30-fold higher activity than the prototype.

## Discussion

HPV16 intratypic variants have been extensively studied. It has been proposed that HPV16 variants with E6 sequence variation are most closely related to the risk factor for development of squamous intraepithelial lesions and invasive carcinoma [14]. In the present study, HPV16 prototype (18.3%) and the 5 different HPV16 variant sub-lineages, As (61%), AA1 (8.5%), EUR (7.3%), AFR2 (3.7%) and J135C (1.2%), were detected (Table 1). The HPV16As detection rate increased according to the severity of the lesion, 30% of LSIL, 63.9% of HSIL and 66.7% of SCC. This result shows that HPV16As infection is associated with a high risk of HSIL and SCC development with an odds ratio of 4.387; 95% CI 1.043-18.457; *P* = 0.042 compared to prototype and other sub-lineages. Moreover, in comparison to HPV16 prototype, this result shows an increased association of HPV16As with risk for cervical cancer. This study suggests that HPV16As is an oncogenic risk for cervical cancer progression.

A study of HPV16 variants in Khon Kaen, Thailand found HPV16As in 73.9% of HPV16-positive cervical cancer samples and showed a risk association with CIN II-III and SCC [12]. Our previous and present studies confirmed the strong association of HPV16As with cervical cancer development in Thai women. Some studies have shown that infection with HPV16 prototype is associated with a lower risk in progression to cervical cancer than that caused by other variants. Sequence variation among HPV16 variants may influence the event of HPV persistence and progression to CIN and cervical carcinoma [9,15].

Using the HPV16 prototype [GenBank:AY686584] as a reference sequence, we detected a total of 38 nucleotide variation positions in the LCRs of 43 HPV16As cases. This result agrees with previous reports of a HPV16As-specific nucleotide variation in the LCR at position 7842 [16-18]. At this position (7842), the majority of the LCR samples had a nucleotide change from G>A (90.7%), whereas the remaining samples had a G>T change. We also identified additional nucleotide variations at positions 7175A>C, 7177 T>C, 7193 G>T, 7201 T>C, 7287A>C, 7521 G>A, 7730A>C, 24C>T and 81 G>T, which were found in all (100%) samples. Other common variations were 7270C>T (95.3%) and 7289 A>T (95.3%). These sequence variations may be typical of the LCR from HPV16As in this region (Table 2).

In this study, 10 novel nucleotide variations, which were previously unreported in the literature, were found in HPV16As LCRs (Table 2); however, two of them were found in only one sample (7617C>A and 7844A>C) and may have occurred by PCR amplification. These variations were associated with YY-1 binding sites, and among them, a deletion or substitution was found to enhance early promoter transcription. It was suggested that mutation affecting YY1-motifs in the LCR is one of the mechanisms that enhance viral oncogene expression during the course of cancer cell progression [19]. Additionally, several studies have reported that cellular factors, such as AP-1, GRE, NF-1, NF-IL6, OCT-1, SP-1, TEF-1, TEF-2 and YY-1, either stimulate or inhibit p97 promoter activity [20-22]. Therefore, these variations could be related to the early promoter activation of HPV16As. With respect to positions 7429 G>A, 7874C>G, 28 G insertion with A, 46 T insertion with G and 61 T insertion with G, these mutations are located close to E2BS-4 (nt 7453–7464), E2BS-3 (nt 7860–7871), SP-1 and E2BS-2 (nt 35–46) and E2BS-1 (nt 50–61), respectively [11]. These novel nucleotide variations in the LCR of HPV16As may play a crucial role in the transcriptional modulation of the HPV16 E6 and E7 oncogenes via the p97 promoter. Lace et al. [7] reported that p97 promoter activity of CAT reporter containing different LCR mutation in HeLa cell line. The results showed that transcriptional activity of HPV16As LCR variations was higher than that of the HPV16 prototype.

The HPV16As isolated from samples no. 15 (HSIL case) and 36 (SCC case) with common nucleotide variation in the LCR (Tables 2 and 3) showed an approximate 5-fold increase in p97 promoter activity compared to the prototype. In accordance with previous results [13], EUR and AA1 transcriptional activity studied in the present study also show higher activity than prototype. This study also shows higher activity of EUR than common HPV16As.

Interestingly, the activity of AA1 has similar patterns to the LCR from the HPV16As-sv1 and HPV16As-sv14 which contained novel variations proximal to the p97 promoter that showed transcriptional activity with 19 and 30-fold higher activity than the prototype. These data suggest that novel nucleotide changes at 7429 G>A and 7874C>G, which are proximal to p97 (at positions 28, 46 and 61) in the LCRs from HPV16As, are associated with high transcriptional activity. Therefore, the oncogenic potential of HPV16As could be influenced by specific sequence variation, especially at positions that are proximal to the promoter region in the HPV16 LCR.

Veress et al. [10] reported transcriptional activity of LCRs from AA HPV16 variants by luciferase assay in C33A cells. The results showed a 1.7-fold increase in transcriptional activity with the AA isolate LCR and a very similar transcriptional activity with the EUR LCR compared to that with the LCR reference. This increased activity of the AA isolate could be attributed to nucleotide changes found at the 3’ end of the LCR (nt7660-7890).

In addition, in 2001 Veress et al. [13] showed that the transcriptional activity of HPV16 full-length AA and EUR isolated from clinical specimens was higher than prototype.

Kammer et al. [11] reported that 3.3- and 2.8-fold increases in p97 promoter activity were detected in the Asian American c (AAc) and North American 1 (NA-1) variants, respectively, when compared with the European reference clone. The region in the AAc and NA-1 variants that is responsible for enhanced transcription could be the E6-proximal end of the LCR (nt 7619–124). Similar results were obtained by Veress et al. [13], who showed that the enhanced transcriptional activity of the AAc variant was due to nucleotide changes in the 3’ region of the LCR. However, a deletion variant lacking the whole enhancer and both silencer regions, either the YY-1-specific silencer alone or together with the CDP-specific silencer, retained substantial enhancer activity on the p97 promoter. Chen et al. [23] studied mutations in the LCR and their functional consequences in oral cancer. They found that promoter activity of the mutated HPV16 LCR was significantly higher than that of the wild type HPV16 LCR, suggesting that mutations in the LCR of HPV in oral cancer leads to increased expression of the E6/E7 oncoproteins, which might contribute to the carcinogenic process.

## Conclusion

Several nucleotide sequence variations were found in the LCR of HPV16As and showed specific patterns in Thai women. The LCR polymorphisms of HPV16As contribute to the alternative mechanism involved in HPV16 oncogenicity and demonstrate a correlation between HPV16 infection and progression to cervical cancer in Thai women. Therefore, our study provides important information concerning the biological significance of intratype genomic variability of HPV16, which ultimately could be used to control these infections.

## Methods

### Samples

A total of 82 samples of HPV16 positive DNAs were selected from our previous study [24] that was performed with 410 archival formalin-fixed paraffin-embedded (FFPE) cervical tissues of Thai women. The samples were used for a study that purposed to investigate the function of HPV16 variants, E2 and LCR polymorphism, and is approved by the Khon Kaen University Ethics Committee for Human Research, as per the Helsinki Declaration with reference number HE500813. The DNA samples were extracted from cervical tissues that were histologically diagnosed as low-grade squamous intraepithelial lesions (LSIL, 10 cases), high-grade squamous intraepithelial lesions (HSIL, 36 cases) and squamous cell carcinoma (SCC, 36 cases).

### HPV16 variant determination

DNA from the selected cases was used as a template to amplify the full-length HPV16 E6 gene by PCR using 3 primer pairs (Table 4) according to the methods of de Boer et al. [25]. Amplicons were analyzed by 1.5% agarose gel electrophoresis, purified and sequenced using an automated sequencer provided by the Molecular Informatics Laboratory, Hong Kong.

**Table 4:** Primers used for HPV16 E6 gene and HPV16 LCR amplification

**Primers**	**Sequences** (**5**’ **to 3**’)	**Positions**	**Product size** (**bp**)
E6-1 F	TTGAACCGAAACCGGTTAGT	nt 46–65	211
E6-1R	GCATAAATCCCGAAAAGCAA	nt 237–256	
E6-2 F	GCAACAGTTACTGCGACGTG	nt 204–224	235
E6-2R	GGACACAGTGGCTTTTGACA	nt 419–438	
E6-3 F	CAGCAATACAACAAACCGTTG	nt 371–391	220
E6-3R	TCATGCAATGTAGGTGTATCTCC	nt 568–590	
LCR1-F	GAAAACGAAAAGCTACACCCA	nt 7083-7104	286
LCR1-R	CAATGAATAACCACAACACAATTA	nt 7345-7368	
LCR2-F	GCTTGTGTAACTATTGTGTCATG	nt 7289-7311	292
LCR2-R	GTGCAGGTCAGGAAAACAG	nt 7562-7580	
LCR3-F	ACTTGTACGTTTCCTGCTTG	nt 7525-7544	350
LCR3-R	GTGTAACCCAAAATCGGTTTGC	nt 7853-7874	
LCR4-F	GTCACCCTAGTTCATACATGA	nt 7777-7797	231
LCR4-R	TGCAGTTCTCTTTTGGTGC	nt 85-103	

### Analysis of HPV16As LCR polymorphisms

Forty-seven DNA samples from the HSIL and SCC cases were classified as HPV16As and investigated for LCR polymorphisms.

Four primer pairs were designed for LCR sequence amplification (Table 4). A 50 μl PCR mixture consisted of 10x PCR buffer, 2.5 mM MgCl_2_, 10 mM dNTP, 1 U Taq DNA polymerase, 10 pM specific primers and 3 μl of DNA template. PCR products were analyzed by 1.5% agarose gel electrophoresis and sequenced by the Molecular Informatic Laboratory, Hong Kong. Nucleotide sequences of amplified LCRs were aligned and compared with the HPV16 reference sequence [GenBank:AY686584], using the bioinformatics genomic tool, Multalin. The HPV16 prototype used in this study was obtained from an HPV16 reference plasmid (kindly provided by Prof. Dr. Ethel-Michele de Villiers), and the sequence was confirmed to correspond with AY686584, which is available through the GenBank database.

### Plasmid construction

Four samples of HPV16As and the sub-lineages, AA1 and EUR, from SCC cases that were found in this study were selected for plasmid construction. Two samples (no. 15 and no. 36) have nucleotide variations in the LCR that are commonly found in HPV16As. The other two (no. 30 and no. 42) have different nucleotide variations in the LCR sequence (Table 2 and 3). The LCR of the HPV16 reference plasmid was also used for plasmid construction to compare the transcriptional activity.

Full-length LCRs containing the HPV16 promoter, p97, for oncogenes E6 and E7 were amplified by PCR using forward LCR1 and reverse LCR4 primers (Table 4). The amplified full-length LCR PCR product was ligated into the pDRIVE cloning vector (Qiagen, Hilden, Germany) and subcloned into the promoterless luciferase reporter vector, pGL3-Basic (Promega, Madison, WI, USA). The constructed vectors were verified by sequencing.

### Transcriptional activity analysis

The pGL3 vectors containing LCRs from HPV16 prototype, four samples of HPV16As, one sample of AA1 and one sample of EUR were used for transcriptional analysis. C33A, a human cervical cancer cell line, was transiently transfected with 0.8 μg of the plasmid vectors using Lipofectamine 2000 (Invitrogen Life Technologies, Carlsbad, CA, USA) according to the manufacturer’s instructions. The cells were harvested at various times (12, 24 and 48 hours) after transfection to optimize the activity. The transfected cells were analyzed by the Bright-Glo™ luciferase assay reagent (Promega, Madison, WI, USA). Luciferase activity was determined using a Modulus Single Tube Multimode Reader (Turner Biosystem). The pSV-β galactosidase control vector (Promega, Madison, WI, USA), which contains the SV40 early promoter and enhancer upstream of the Lac Z gene, was used to control for transfection efficiency and luciferase activity.

### Statistic analysis

The Chi-square test was used to analyze the correlation between the HPV16 variant sub-lineages and cervical cancer. Student’s t-test was used to analyze the transcriptional activity of the HPV16 prototype and HPV16As. A *P* value of less than 0.05 was considered as statistically significant. The risk of HPV16As that associated with different grade lesions was analyzed using odds ratio (OR) and 95% confidence interval (CI). The analysis was performed with a SPSS16 program.

## Abbreviations

HPV: Human papillomavirus; LCR: Long control region; HPV16As: HPV16 variant sub-lineage Asian; EUR: European; AA1: Asian-American 1; AFR2: Asian African-2; J135C: Java-135C; As-sv: HPV16As sub-variant; LSIL: Low-grade squamous intraepithelial lesion; HSIL: High-grade squamous intraepithelial lesion; SCC: Squamous cell cervical carcinoma; TEF-1: Transcription factor binding site; GRE-1: Glucocorticoid response element; YY-1: Yin yang factor; SP-1: *Trans*-acting transcription factor; Oct-1: Octamer binding transcription factor; E2BS: E2 binding site.

## Competing interests

The authors declare no competing interests.

## Authors’ contributions

CP and BK were responsible for the study design. PK, SS, KT and CS conducted the sample recruitment process. PW, PS, CP and TE were responsible for the molecular biology work. CP, BK and TE participated in the interpretation of the data and drafted the manuscript. All authors read and approved the final manuscript.
